# Automated Nanoliter Volume Assay Optimization on a Cost-Effective Microfluidic Disc

**DOI:** 10.1021/acs.analchem.4c04210

**Published:** 2024-12-28

**Authors:** Renna L. Nouwairi, Carter K. Jones, Maura E. Charette, Emilee Holmquist, Zoey Golabek, James P. Landers

**Affiliations:** † Department of Chemistry, University of Virginia, Charlottesville, Virginia 22904, United States; ‡ Department of Mechanical Engineering, University of Virginia, Charlottesville, Virginia 22904, United States; § Department of Pathology, University of Virginia, Charlottesville, Virginia 22904, United States

## Abstract

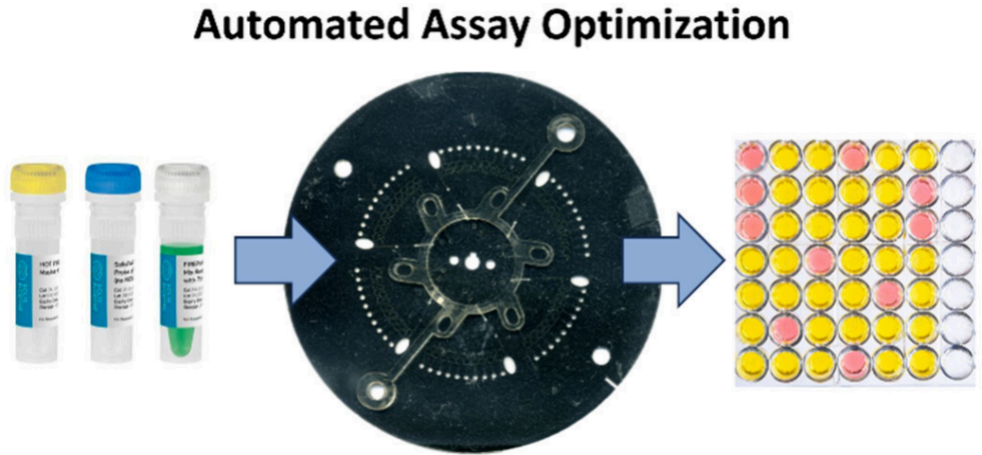

Optimizing multireagent assays often requires successive titration of individual components until the optimal combination of conditions is achieved. This process is time-consuming, laborious, and often expensive since parallelized experimentation requires bulk consumption of reagents. Microfluidics presents a solution through miniaturization of standard processes by reducing reaction volume, executing multiple parallel workflows, and enabling automation. While single-digit microliter reactions can be effective, scaling to nanoliter volumes without employing droplets is difficult. We describe a cost-effective, customizable centrifugal microdisc for optimizing assays pertinent to a broad array of applications. An automated two-stage metering process leverages tunable, laser-actuated valves that retain defined fluidic volumes upon opening and meter discrete nanoliter volumes into downstream architecture. We demonstrate that ∼150 nL volumes could be metered and tuned for specific applications. We illustrate the potential for controlled metering of up to four reagents with high parallelization for rapid, cost-effective assay optimization with minimal manual intervention.

Since the advent of the miniaturized total analysis (μTAS) concept by Manz and colleagues in the late 1980s,^1^ we have witnessed an evolving capability for performing chemical assays at ever-decreasing volumes. Executing chemistry at the microliter scale has been eclipsed by microfluidic systems that work effectively in the nanoliter,^2,3^ picoliter,^4,5^ and even femtoliter^6,7^ regimes. However, reports on the manipulation of microliter and nanoliter volumes with combinatorial capability have not been described. This is a critical part of chemical and biochemical assay optimization, where the stepwise change in concentration of any number of reagents must be methodically trialed to identify the ideal conditions for the most effective reaction. For a simple biomolecular chemical reaction, this might be viewed as trivial; for example, when determining the optimal analytical range for visual detection of a drug,^8^ the colorimetric indicator concentration is held constant while the target analyte is titrated over a concentration range that could be an order of magnitude to define the color (e.g., RGB, HSB color space) for optimal visual detection. With reactions of a higher order, this becomes exponentially more complex as a multitude of reagent combinations and permutations be tested, leading to a labor-intensive, time-consuming, and potentially expensive optimization process.^9−11^

Quake’s group was the first to show that microfluidics was uniquely poised to address this with minimal manual interaction, applying it directly to protein crystallization and demonstrating the benefit for optimizing reactions in the micro- and nanoliter regime.^12^ Chemical reaction optimization in much smaller volumes has numerous benefits, including the ability to enhance reaction kinetics by minimizing diffusion and, if needed, amend microscale architecture for parallelization and automation of routine lab workflows with minimal manual intervention.^13^ In terms of nanovolume metering in microfluidic systems, droplet microfluidics enables creation of nano- and even picoliter “reactors”, but analysis of the reaction products typically requires interrogation “*in situ”* using sophisticated analytical systems (e.g., fluorescence or mass spec).^14,15^ However, there are more conventional means for carrying nanoliter metering and mixing, including centrifugal microfluidics.

Centrifugal microdevices include capable valving methods, including passive valves that rely on passive forces (e.g., pressure, hydrophobic barriers, etc.) and active valves that require external interventions (e.g., heat or laser irradiation), that can combined with judiciously designed architectural features to facilitate fluid transport, mixing, metering, and more.^16,17^ In single-stage metering, fluid is aliquoted into a metering chamber(s) to a specified volume and excess fluid overfills to a waste chamber. Two-stage metering performs the same initial single-stage method, but the metered volume can only connect with subsequent fluidic architecture after passing through a valve.^18^ With carefully designed microfluidic architecture, these unit operations can be employed to split fluid into sub-1 μL volumes and perform multiple reactions in parallel.

Passive valves have been incorporated into micro-, nano-, and even pico-fluidic devices, as they are simpler to incorporate compared to active valves.^19^ Andersson et al. developed a centrifugal microdisc that implemented capillary action to split a volume of fluid into smaller aliquots (stage one) and then into hydrophobic valves (stage two) that could be overcome by centrifugal force, to meter 20–200 nL into parallel reaction architectures.^2^ While this disc was designed to reproducibly perform an impressive 100 reactions simultaneously, fabrication requires complicated and laborious processes, and the hydrophobic barriers were made via local surface modifications that require complex spatial precision and volume deposition. Alternatively, Mark et al. designed a centrifugal PDMS microdisc with a two-stage aliquoting process that incorporated a centrifuge-pneumatic valve, which enabled fluid to flow when the rotational force overcame the counter-pressure of the subsequent channel, to meter 1–36 μL of fluid.^18^ While notable, this device’s working range does not extend into the nanoliter regime. More recently, Morikawa et al. developed a glass device for handling picoliter volumes by designing picoliter-sized chambers with strategic geometry to induce capillary pinning, which leverages a large contact angle to prevent fluid flow.^4^ In these examples, the fine architectural features capable of metering and manipulating nanovolumes required costly substrates that were fabricated via lithography, a laborious, time-consuming, and expensive technique.^20^ While these microdevices could be modified to perform parallel titrations for assay optimization, the cost of the device would likely negate cost saved in terms of reagents, and the fabrication methods required are not widely accessible or ideal for rapid prototyping.

Previously, we described the Print, Cut, and Laminate (PCL) fabrication method,^21^ which is amenable to rapid prototyping of cost-effect microdevices, consumes less than 30 min, and only requires a laser cutter and standard office laminator to impart architectural features onto inexpensive polymeric materials. Devices created with the PCL method leverage an active valving method based on Garcia-Cordero’s optically addressable microvalves,^22^ but our microdiscs incorporate an optically dense layer of polyethylene terephthalate (PeT) to create normally closed valves that can be opened and subsequently closed via laser irradiation to connect and disconnect fluidic layers.^23^

Here, we describe exploitation of the PCL method and these laser-actuated valves as a metering mechanism for submicroliter volumes of fluid and explore how it can be multiplexed for parallel, cost-effective, and automated assay optimization. The rotationally driven microdisc incorporates a two-stage metering technique. By tuning the valving strategy (e.g., valve shape, laser irradiation position, and metering architecture), the volume of fluid released to downstream architecture can be carefully controlled. The architectural details were subsequently refined for device use for titration of a drug standard into a colorimetric indicator and further customizability of the microdevice was demonstrated. This proof-of-concept device leverages the combination of nanoliter fluidic control with an accessible fabrication process and permits assay optimization with a tunable microdevice, requiring very minimal manual intervention thanks to careful consideration of valving strategy and a corresponding external platform that enables automation of on-disc operations.

## Experimental Section

### Microfluidic Device Fabrication

The centrifugal microfluidic discs described herein were constructed using the Print, Cut, and Laminate (PCL) fabrication method.^21^ Briefly, a CO_2_ laser (VLS3.50, Universal Laser Systems, Scottsdale, AZ, USA) ablated architectural features designed in AutoCAD (2019, AutoDesk Inc., San Rafael, CA, USA) into thermoplastic substrates. The core disc was comprised of five layers of 101.6 μM polyethylene terephthalate (PeT) films (Film Source, Inc., Maryland Heights, MO, USA). The outer layers (layers 1 and 5) enclose the microdevice, while inner fluidic layers (layers 2 and 4) were coated in a heat sensitive adhesive (HSA; Adhesives Research, Inc. Glen Rock, PA, USA) and separated by an optically dense black PeT (bPeT) layer (layer 3) (Lumirror* X30, Toray Industries, Inc., Chuo-ku, Tokyo, Japan) to permit laser-actuated valving.^22,23^ The individual layers were aligned and passed through an office laminator (UltraLam 250B, Alikes Products Inc., Mira Loma, CA, USA) to create the core device. Laser-cut poly(methyl methacrylate) (PMMA; 1.5 mm McMaster Carr, Elmhurst, IL, USA) accessory pieces were bonded to the microdevice using pressure sensitive adhesive (PSA; Arcare 7876, Adhesives Research Inc.) to increase select chamber depth (volume).

### Mechatronic Spin System

The custom-built spin system used to generate centrifugal force and perform laser-actuated valving, termed the Power, Time, and Adjustable z-Height Laser (PrTZAL), is detailed elsewhere.^23^ A brushless DC motor (Digi-Key Electronics, MN, USA) with a 3D-printed disc mount was connected to a linear motorized translational stage (MTS50-Z8, ThorLabs) that moved in the *x*-direction (Figure S1A). A 700 mW 638 nm laser diode (L638P700M, ThorLabs, Inc., Newton, NJ, USA) focused with a collimation tube containing a single aspherical lens element (LTN330-A, Thorlabs, Inc., Newton, NJ, USA) was positioned above the disc. Targeted microvalves were located and opened by adjusting two variables: the radial distance from the center of rotation (d) and the angle of rotation (θ) relative to a homing notch (Figure S1B). The positioning of the mounted microfluidic disc relative to the laser was adjusted radially though the translational stage that moved the microdevice in the *x*-direction with a resolution of 0.04 mm, and the angular positioning was adjusted through the brushless DC micromotor with a 0.2° resolution, an embedded homing notch, and a photointerrupting optical switch (TT Electronics/Optek Technology, Woking, UK). Normally closed valves were opened via laser irradiation (500 mW, 500 ms) by positioning the laser 15.00 mm above the valve (Figure S1C). All functions in the PrTZAL system were operated by a 32-bit multiprocessing microcontroller (Propeller P8 × 32A-M44; Propeller Inc., Rockland, CA, USA).

To evaluate the accuracy and precision of the PrTZAL system, rectangular valves were opened once or 10 consecutive times at the center of the valve (d = 34.4 mm), and the disc was imaged using an Epson Perfection V100 Photo desktop scanner (Seiko Epson Corporation, Suwa, Nagano Prefecture, Japan). The radial and angular accuracy/precision was evaluated by opening valves 10 times at different radii (d = 33.7, 34.4, 35.1 mm) or at angles that differ ±1° from the center of the valve.

### Optical Analysis of Fluidic Architecture

Characterization and optimization of microfluidic architecture was performed through on-disc fluidic studies using 0.01 M allura red in 1X TE buffer for visualization. Discs were imaged with the Epson scanner, and images were analyzed using the Fiji distribution of ImageJ software. Circular regions of interest (ROIs) within each detection chamber were selected following the ‘Crop – Threshold–and – Go’ method described by Woolf et al.^24^ Upon selection of the targeted region, the pixel count was measured. Pixels outside of the circular 90 × 90 pixel ROI centered over the detection chamber were cleared and the color threshold was set to an L*a*b* colorspace with the following thresholding parameters: L* = 1–114 pass, a* = 141–255 pass, and b* = 0–255 pass. A calibration curve correlating pixel count to volume was established by pipetting 100–500 nL in 100 nL increments, of the allura red dye into detection chambers and measuring the number of pixels associated with the known volume. The resultant linear trendline (y = 5948.5x + 293.7; R^2^ = 0.9916) was used to determine volume by inputting the measured pixel count for y and solving for x (nL volume).

For hue analysis, the ‘Crop–and – Go’ method^24^ was implemented whereby the raw images of the circular 60 × 60 pixel ROI centered over the detection chambers were converted to a hue-saturation-brightness (HSB) stack to enable hue measurement.

### Characterization of Dual-Stage Metering Workflow

Operation of the microfluidic disc required pipetting 12 μL of 0.01 M allura red dye in 1X TE buffer into the reagent chamber. Following laser-actuated valve opening of the valve directly below the reagent chamber, dye was rotationally driven into the metering channel and valves (3000 rpm, 30 s). The arrayed valves were then opened at specific radii corresponding to the top, center, or bottom of the valve (d = 33.7, 34.4, or 35.1 mm, respectively), and fluid was metered into the detection chambers upon spinning at 3000 rpm for 30 s. Clockwise (CW) and counterclockwise (CCW) rotation was achieved by inputting 3000 rpm or −3000 rpm into the PrTZAL system, respectively. Alternatively, valves were opened at specific angles corresponding to the left, center, or right of the valves (measured in AutoCAD) prior to metering fluid into the detection chambers.

### Colorimetric Detection and Titration of Illicit Drug

#### Preparation of Drug Sample and Reagents

Cocaine standard (1 mg/mL, C-008, Cerrilant Corporation, Round Rock, Texas, USA) stored in acetonitrile was vacufuged and reconstituted to 10 mg/mL by aliquoting 25 μL of the standard into 20 0.2 μL PCR tubes (500 μL total). Standards were protected from ambient light exposure during preparation. The 20 PCR tubes were nested in 1.5 mL tubes and placed in a vacufuge (Eppendorf Vacufuge 5301, Eppendorf, Hamburg, Germany) for 25 min at room temperature to remove the organic storage solvent. Upon removal from the vacufuge, samples were placed on ice in a light-tight box, individually reconstituted in 2.5 μL hydrochloric acid (HCl, 10 M; Thermo Fisher Scientific, Waltham, MA, USA), and all 20 PCR tubes were combined for a 50 μL stock solution of cocaine standard with a final concentration of 10 mg/mL.

The cobalt thiocyanate colorimetric indicator, known as Scott’s Reagent, was prepared according to a protocol described elsewhere.^25^ Briefly, 2.0 g cobalt(II) thiocyanate was dissolved in 10% acetic acid solution (v/v. acetic acid:water). Hydrochloric acid was diluted to 0.1 M in water. Dye studies were done with standard food dye added to water.

#### Dye Study

The proof-of-concept disc design described previously was adjusted to contain a second array of metering architecture to accommodate two different reagents in 15 separate detection chambers. A mock titration was done using water containing either blue, red, or yellow food dye. All were pipetted into the disc, with 2 μL yellow dye into each detection chamber and 15 μL of red and blue dye into the upper and lower reagent chambers, respectively. Upon opening the valves directly under the reagent chambers, the disc was spun at 3000 rpm for 30 s to fill the metering architecture. Valves were opened at the top, middle, or bottom of the valve using 33.7 mm, 34.4 mm, or 35.1 mm as the respective radii for the upper array, or using 43.6 mm, 44.3 mm, or 45 mm for the lower array radii, respectively. After opening valves in the lower array containing blue dye, the disc was spun at 3000 rpm for 10 s CW and CCW to facilitate mixing in the detection chamber. The disc was imaged on the aforementioned Epson desktop scanner before opening valves to add red dye to the detection chamber. Upon spinning CW and CCW at 3000 rpm for 10 s to add red dye into the detection chambers, the disc was scanned, and all images were analyzed using the Fiji distribution of ImageJ software. Hue was objectively measured via the ‘Crop–and – Go’ method,^24^ in accordance with the above optical analysis protocol.

#### Illicit Drug Titration

A titration of drug concentrations was conducted by pipetting 2 μL of cobalt thiocyanate (Thermo Fisher Scientific, Waltham, MA, USA) into each detection chamber. Fifteen μL of 0.1 M HCl and 15 μL of 10 mg/mL prepared cocaine standard were added to the upper and lower reagent chambers, respectively. Upon opening the reagent chamber valves, the disc was spun at 3000 rpm for 30 s to fill the metering architecture. Valves were opened at predefined radii corresponding to the top (upper array: 33.7 mm; lower array: 43.6 mm), middle (upper array: 34.4 mm; lower array: 44.3 mm), or bottom of the valve (upper array: 35.1 mm, lower array: 45 mm) to meter select volumes of reagents in the detection chamber. After opening all valves, the disc was spun CW and CCW at 3000 rpm for 10 s to facilitate mixing in the detection chamber. The disc was imaged on an Epson Perfection V100 Photo desktop scanner and images were analyzed using the Fiji distribution of ImageJ software to measure hue in the detection chambers following the protocol described above with an extra step: the hue wheel was rotated 127 to avoid splitting the red between the color spectrum.^24^

### Expanded Nine-Layer Microdisc

#### Device Fabrication

The microfluidic disc was adapted to contain four additional core layers, nine in total, to increase the available fluidic architecture and accommodate titration of four reagents. This microdisc was fabricated via the PCL method using 101.6 μM PeT films (Film Source, Inc., Maryland Heights, MO, USA). The outer layers (layers 1 and 9) enclosed the disc. The inner fluidic layers (layers 2, 4, 6, and 8) were coated with HSA (Adhesives Research, Inc., Glen Rock, PA, USA) to bond layers together; these layers were separated by a layer of optically dense bPeT (Lumirror* X30, Toray Industries, Inc., Chuo-ku, Tokyo, Japan) (layers 3 and 7) to permit valving. An additional middle layer of clear PeT (layer 5) separates architecture on the front and back of the disc, so the reagents from the front flow through layers 2 through 4, while the reagents from the back flow through layers 8 through 6. Layers 3 through 7 were aligned and passed through an office laminator (UltraLam 250B, Alikes Products Inc., Mira Loma, CA, USA) prior to curing in an oven (BINDER GmbH, Tuttlingen, Germany) at 40 °C with 2.83 psi for 1 h. Afterward, layers 1, 2, 8, and 9 were aligned to the top and bottom of the disc, and the device was passed through the office laminator again. The bonded nine-layers were placed in the same oven at 40 °C and 2.83 psi for 16 h. Laser-cut PMMA accessory pieces were bonded to the top and bottom of the microfluidic disc using PSA (Arcare 7876, Adhesives Research Inc.) to increase the depth and volume of the reagent chambers.

#### Dye Study

A dye study was run on the nine-layer microdevice using water containing either green, blue, yellow, or red dye. Water (2 μL) was pipetted directly into each of the 15 detection chamber, while 20 μL of blue and green dye were pipetted into the upper and lower reagent chambers on the front of the disc, respectively, and 20 μL of red and yellow dye were pipetted into the upper and lower reagent chambers on the back of the disc, respectively. The valves directly under the reagent chambers on the front of the disc were opened, and the blue and green dye were metered across the fluidic architecture by spinning the disc CW at 3000 rpm for 10 s. Similarly, valves under the reagent chambers on the back of the disc were opened and the disc was spun CCW at 3000 rpm for 10 s to fill the metering channels with yellow and red dye. Valves containing green dye in the front lower array were opened at the top, middle, and bottom of the valves using radii values of 43.6, 44.2, and 44.8 mm, respectively. The microfluidic disc spun CW and CCW at 3000 rpm for 10 s to drive the green dye into the detection chambers and facilitate mixing between the water and dye. The disc was imaged on an Epson Perfection V100 Photo desktop scanner (Seiko Epson Corporation, Suwa, Nagano Prefecture, Japan) before opening the valves containing blue dye in the front upper array. The radii values used for the top, middle, and bottom of the upper array valves were 33.7, 34.3, and 35 mm, respectively. The disc was spun CW and CCW at 3000 rpm for 10 s to mix the blue dye in the detection chambers, imaged, and then flipped to expose the architecture on the back of the disc. The same radii values for the upper and lower arrays on the back of the microdevice containing red and yellow dye, respectively, were used. The valves with yellow dye were opened, the disc was spun CW and CCW at 3000 rpm for 10 s, and the disc was imaged. The same opening, spinning, and imaging process was used for the upper array on the back containing red dye. All images were analyzed using the Fiji distribution of ImageJ software. The raw images were converted to a hue-saturation-brightness (HSB) stack to permit an objective hue measurement via the ‘Crop–and – Go’ method. Because of the extra layers in the nine-layer NMD, the ROI was changed to a circular 40 × 40 pixel area.

### Statistical Analysis

Statistical analysis was carried out using Microsoft Excel. Data was analyzed by calculating averages and standard deviations. *P* values of >0.05 were considered not significant for all T-Tests and one-way or two-way analysis of variance (ANOVA) performed.

## Results and Discussion

### Microdisc Design and Operation

**Figure 1:**
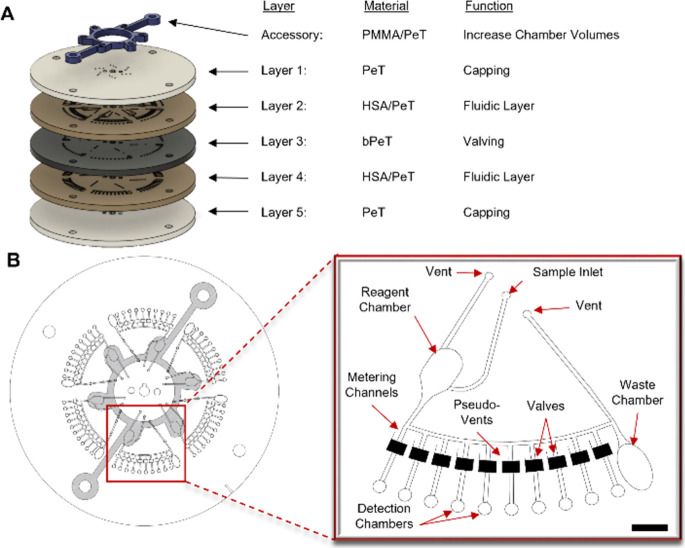
Microfluidic architecture for the rotationally driven nanoliter metering disc. (A) Exploded view of five-layer disc consisting of polyethylene terephthalate (PeT; layers 1 and 5), fluidic layers (layers 2 and 4) comprised of PeT with a heat-sensitive adhesive (HSA) on both sides; and a separation layer of black PeT for laser-actuated valving. The accessory pieces, consisting of poly(methyl methacrylate) (PMMA) capped with PeT, are attached to the five-layer disc via a pressure-sensitive adhesive (PSA). (B) The centrifugal microfluidic disc containing six domains as viewed from the top. The inset depicts a single domain. Scale bar, 5 mm.

The prototype nanoliter metering disc (NMD) shown in Figure 1 was fabricated via the previously described PCL method,^21^ which allows for complete device fabrication and assembly in under 30 min and at an average estimated cost of ∼ $1 USD/disc. Each microfluidic device (roughly the size of a standard compact disc) is composed of 5 core layers of laser ablated polymeric materials, including clear polyethylene terephthalate (PeT), an optically dense black PeT (bPeT), and a heat sensitive adhesive (HSA) for layer bonding (Figure 1A). PeT layers 1 and 5 served as capping layers, with layer 1 containing sample inlet ports and vents to ensure unimpeded flow. Layers 2 and 4 are composed of HSA-flanked PeT and contain the microfluidic architecture. The bPeT layer (3) is critical for active valving, whereby a red laser ablates an ∼80 um pinhole in a normally closed sacrificial valve, thus, providing a fluidic connection between architecture in layers 2 and 4. The prototype mechatronic/laser ablation system, also known as the Power, Time, Z-Height Actuated Laser (PrTZAL) system,^23^ that interfaces with the NMD to facilitate valving are detailed in Figure S1. Accessory pieces, made from poly(methyl methacrylate) (PMMA) and capped with PeT, are bonded to the disc through a pressure sensitive adhesive (PSA) to increase chamber volume capacities. Architecture in one representative domain of six identical domains is highlighted in Figure 1B, which includes a reagent chamber, a metering channel that feeds into the laser-actuated valves, a waste chamber for reagent overflow, and detection chambers.

Initial characterization of the fluidic workflow was performed using dye studies (Figure S2). The disc was designed to leverage two-stage metering with active valving, whereby at least 10 μL of fluid was manually pipetted into the reagent chamber and rotationally driven into a connected network of 10 downstream metering channels containing corresponding sacrificial valves. Upon device rotation, each valve theoretically holds ∼500 nL of fluid, with an additional 200 nL being held in the upstream channel; any excess reagent was driven to the waste chamber. Stage 2 metering involves laser-ablating the sacrificial valves open, then rotationally driving fluid into downstream detection chambers (Figure S2C). The rectangular valves were opened in the center through an automated alignment/ablation process, whereby the radial distance and angular position of the valve was programmed into the corresponding PrTZAL system (Figure S1), along with order of operations and rotational directions (e.g., speed and time).

Multiple architectural features were implemented to ensure reagents flowed as intended. First, valves were positioned at the same radial distance from the center to permit automation of valve opening. This spared the user from manually aligning the laser with each valve, which can consume a significant amount of time and increase issues associated with interoperator variability. Second, the metering channel was tapered to increase fluidic resistance, forcing fluid to fill the metering chambers prior to driving fluid to the waste chamber. Third, a “pseudovent” was added to layer 1 by cutting a 100 μm wide slit to the upper left corner of each vent, allowing fluid to move more easily into downstream detection chambers. Use of a traditional vent (illustrated in reagent and waste chambers in Figure 1B) was not possible because of the narrow proximity of metering architecture; however, the small volume of fluid requires pressure displacement beyond the capability of the system (e.g., below 3000 rpm), thus, a pseudovent was necessary.^16^

Quantitative image analysis characterized this iteration of the metering design, using aqueous red dye for visualization. Scanned images of cropped detection chambers were analyzed for fluid volume using the color thresholding method.^24^ A calibration curve was established to measure the pixel count of red dye in detection chambers containing known fluid volumes from 100–500 nL (Figure S2D).

### Optimization and Characterization of Two-Stage Metering

**Figure 2:**
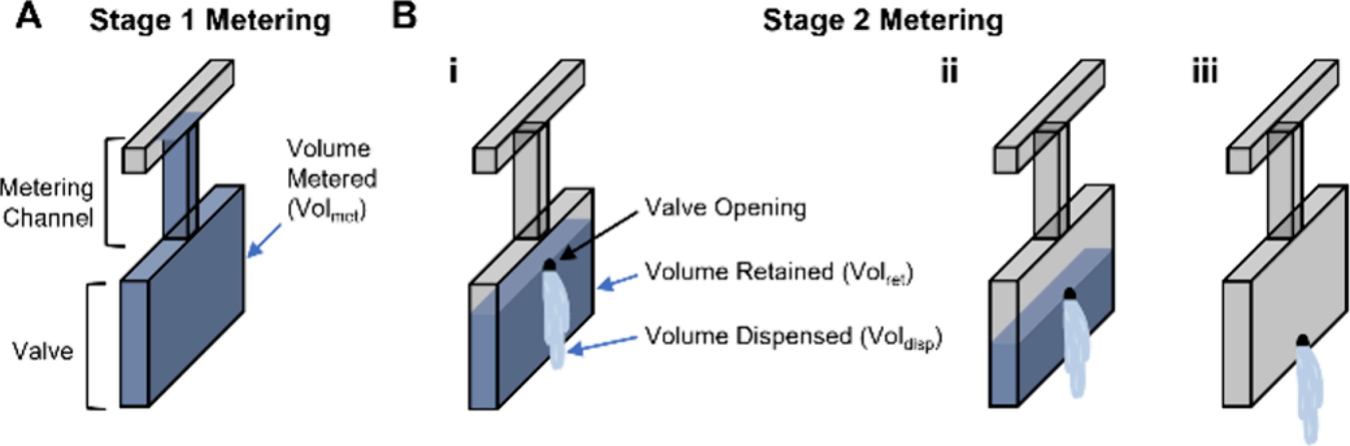
Three-dimensional schematic of the dual-stage metering principal. (A) A fluid filled microvalve after stage 1 metering. (B) Stage 2 metering in which valve is opened via a laser at the (i) top, (ii) center, and (iii) bottom of the valve.

The dual-stage metering theory relies on aliquoting <1 μL into the metering channels and valves in stage 1 and changing the position of the valve opening to tune the fluid volume dispensed into downstream architecture in stage 2. Figure 2 illustrates a valve filled during stage 1 metering (Figure 2A), with stage 2 metering occurring after the valve is opened at the top (Figure 2Bi), center (Figure 2Bii), or bottom (Figure 2Biii) of the valve. Equation formulates this theory: the final volume dispensed from the valve (Vol_disp_) is determined by subtracting the volume retained (Vol_ret_) in the valve postlaser actuation from the initial volume metered (Vol_met_) in stage 1.

It was hypothesized that opening the valve near the top would result in the minor fraction of the total fluid being expelled during disc rotation. Conversely, valving at the bottom would enable maximum expulsion of fluid. With this NMD design, four variables contribute to the reproducibility and metering tunability: (1) the accuracy and precision of the PrTZAL instrument, (2) the valve shape, (3) the location of valve opening, and (4) the architecture upstream of the valve. Each of these variables were evaluated in terms of impact on the fluid volume metered to detection chambers in the second stage, with reproducibility determined by completing ten valving events.

### Instrumental Accuracy and Precision

The preferred valve shape to-date has been rectangular, and the location of the 80 μm ablated valve opening was defined by radial and angular coordinates input into the PrTZAL software. For all of our previous applications, the ablated opening served as a port between fluidic layers, and the position of that ablated hole was always inconsequential, owing to the fact that the volume of fluid traversing the valve was larger (by orders of magnitude) than the valve volume itself. However, when exploiting this system for nanoliter volume metering, the location of the opening matters, as does the volume of valve itself. Since the radial position of the opening determines the volume of fluid dispensed from (Vol_disp_) and retained by (Vol_ret_) the valve, and knowing the first-generation PrTZAL system hardware has inherent resolution limitations (0.04 mm radially and 0.2° angularly),^23^ the reproducibility of repeatedly hitting the same coordinates needed to be evaluated. Holding the opening location constant, valves were opened with either a single firing of the laser or with 10 repeat firings (Figure S3), then imaged. Identical coordinates were input into the PrTZAL system between firings to ensure the system could relocate to the correct position each time. An embedded optical switch assures consistent positioning. Figure S3 illustrates the accuracy and precision of a single and consecutive valve openings in the center of the valve (Figure S3C). To test the radial accuracy, the angular coordinate was held constant but the radial coordinate changed to open the valves at the top, center, and bottom; the angular reproducibility was tested in a similar manner (i.e., left, center, right) (Figure S3D). When comparing the single to the consecutive valve openings, the single fire openings are clean and small, and look dramatically different to holes created with multiple firing. First, repetitive exposure to heat from repeated laser firing caused the size of the hole to expand significantly, although part of the expansion could be from the inherent error in the PrTZAL system hardware. Second, a small, raised ridge, or burr, can be seen around the edge of the larger holes, likely from ablated bPeT debris. While nanoliter volumes were able to be consistently dispensed from concise valve openings after one firing on multiple devices, the consistency of the valve opening shape upon repetitive (n = 10) laser firing is indicative of the high accuracy and high precision of the PrTZAL instrument.

### The Effect of Valve Shape on Metering

**Figure 3:**
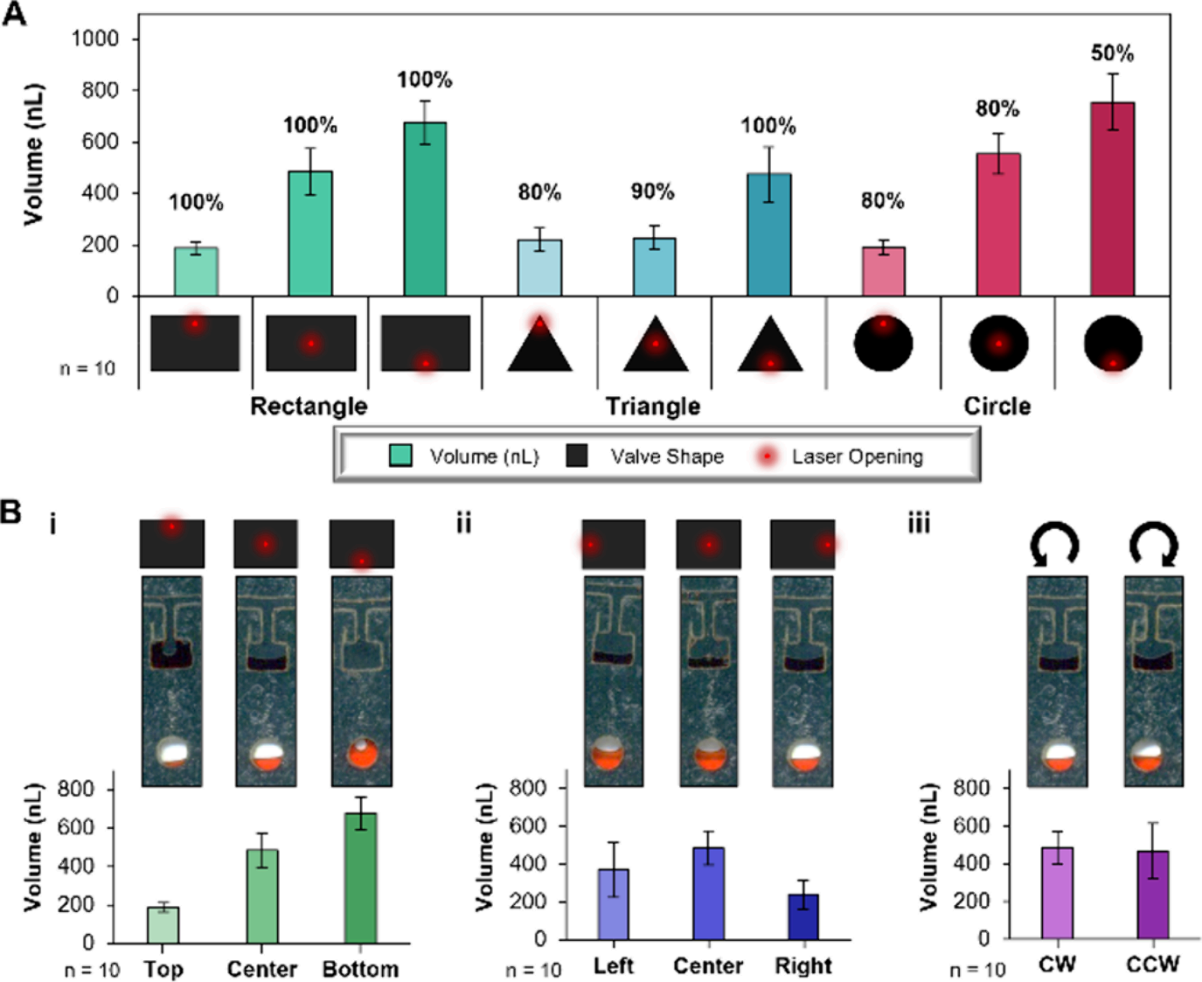
Evaluation of valve shape and opening position on final metered volume. (A) Graphic depiction of the liquid volume dispensed as a result of varying valve shape and opening position; percentages above bars represent the percentage of valves opened (*n* = 10). (B) A comparison of recovered fluid volumes as a result of valve actuation in different (i) radial positions, (ii) angular positions, or (iii) rotational directions.

After examining the repeatability of hitting the same coordinates, we explored the relationship between valve shape and Vol_disp_. Discs were fabricated with 6 domains containing 10 rectangular, triangular, or circular valves per domain that were opened at different radial locations–top, center, or bottom (Figure 3A). For consistency, the dimensions of the metering channel leading to the valve were held constant while the area of the valve was adjusted to ensure the metering chambers retained the same volume (700 nL). In testing, both the Vol_disp_ and the frequency of successful valve openings (%) were evaluated to determine which shape resulted in the smallest volume of fluid metered and the most reproducible valving.

Figure 3A depicts the average Vol_disp_ to the detection chamber across all 10 valves. Generally, as the valve opening is shifted radially downward, the amount of fluid expelled to the detection chamber increases as Vol_ret_ fluid decreases. Depending on the location, the rectangular valves metered an average Vol_disp_ of 190 nL ± 23 nL (top), 480 nL ± 90 nL (center), and 670 nL ± 85 nL (bottom), illustrating a linear increase in metered volume. This was unsurprising given the symmetry of the rectangular shape, which allows the Vol_disp_ to increase by equal amounts when changing the radial position of the laser-ablated hole. Conversely, the triangle valve resulted in 220.0 nL ± 46 nL, 230 nL ± 46 nL, and 470 nL ± 110 nL (top, center, bottom, respectively) of fluid metered. This increase in volume as the valve opening moved toward the bottom was logical since opening the valve at the top versus center does not significantly change the Vol_disp_, but valving at the bottom allows the full volume of fluid to be dispensed. Finally, the circular valve dispensed an average of 190 nL ± 28 nL, 550 nL ± 78 nL, and 750 nL ± 110 nL (top, center, bottom, respectively). The increase (top to bottom) in Vol_disp_ is understandable as the valve width is narrower at the top and bottom relative to that in center; similarly, the Vol_disp_ was not expected to increase significantly when opening at the bottom relative to the center condition. Generally, when the valve is opening at the top position, there is no shape dependence, i.e., no statistical difference in the average volume metered to the detection chamber (one-way ANOVA: p-value = 0.110, α = 0.05) as only the fluid in the channel leading to the valve was dispensed. Conversely, when valves were opened at the lowest radial position, rectangular and circular valves dispensed a similar volume (unpaired T-Test: p-value = 0.140, α = 0.05), but the triangular valve had a lower Vol_disp_; this resulted from fluid being trapped in the acute corners of the valve, a consequence of air expanding as the laser ablated a hole in the valve (Figure S4A). Together, these findings indicate that either the circular or rectangular valves could be used, as they reproducibly dispensed approximately 200, 400, and 600 nL (top, center, and bottom, respectively).

It is worth noting that while the metering chamber held a theoretical volume of 700 nL, a Vol_disp_ of greater than 700 nL was infrequently observed for all valve shapes. One possible cause was the minute volume, estimated as <100 nL, of residual fluid in the metering channel flowing through the open valve. This phenomenon was typically avoided by repeated spinning if residual fluid was seen in the metering channels. Another, more likely, cause of an overestimation of recovered fluid is related to the presence of shadows in the scanned image of the detection chamber, which slightly skew the estimated pixel count during the thresholding portion of image analysis (Figure S4B).^24^ While the latter is unavoidable, as thresholding parameters were set to keep image analysis objective, this increased the standard deviation when averaging Vol_disp_, particularly when larger fluid volumes were estimated to be recovered.

To select a final valve shape, the shape-dependent success rate for valve opening was assessed (Figure 3A). Rectangular valves were the most reproducible with 100% of valves opened at all positions. Triangular valves were less consistent with opening success rates of 80% (top), 90% (center), and 100% (bottom), and circular valves showed the poorest reproducibility with 80% (top), 80% (center), and 50% (bottom). The failure for valve opening generally resulted from slight misalignment of the PrTZAL system due to instrumental error, resulting in either an opening on the valve edge, causing ablated material to block the fluid from the hole, or firing outside of the valve target area entirely. A sub-100% valve opening was not surprising with the triangular valves at the top or center radial positions, or with the circular valves at the top and bottom positions, as those geometries have small angular margins for error compared to the rectangular valve. These results demonstrated that rectangular valves compensate for the limitations of the external laser system, and were selected for all further experiments.

### Radial and Angular Accuracy

Having selected rectangular valves, characterization of the radial and angular accuracy was examined through dye studies by measuring Vol_disp_. As previously discussed, the fluidic Vol_disp_ when opening at the top, middle, and bottom were 190 nL ± 23 nL, 480 nL ± 89 nL, and 670 nL ± 85 nL, respectively (one-way ANOVA: p-value <0.05 × 10^–12^, α = 0.05), and a visual representation depicting one representative example from each valve opening parameter is shown in Figure 3Bi. To assess the angular accuracy of the system relative to the microdevice, rectangular valves were opened in the radial center position and the angle of the valve was adjusted ±1.0° to open the valve in the angular left, center, and right position of the valve (Figure 3Bii). There was no statistical difference in the volume of fluid metered between the left and center conditions (left = 370 nL ± 150 nL; center = 480 nL ± 89 nL; T-Test: p-value = 0.080, α = 0.05) or between the left and right conditions (right = 240 nL ± 76 nL; T-Test: p-value = 0.051, α = 0.05); however, significant variability in the Vol_disp_ was seen when comparing the center and right valve openings (T-Test: p-value = 0.00007, α = 0.05). An increased standard deviation was observed when opening in the left and right positions due to the innate angular error associated with the PrTZAL system. Opening in the center of the valve allowed slightly more fluid to be dispensed as the center of the retained fluid meniscus in the valve aligned with the valve opening. The default counterclockwise direction of rotation from the operating system imparts a Coriolis force^19^ toward the left wall of the valve, ultimately pushing fluid to the left and shifting the meniscus position leftward. This reduces the Vol_disp_ for the right valve opening and contributes to the higher standard deviation observed with the left valve opening. Since the Coriolis force can be significant in centrifugal systems, potentially altering the Vol_disp_, we explored the effect of rotational direction. Results in Figure 3Biii indicate there was no statistical difference based on clockwise (CW) or counterclockwise (CCW) spinning when valves were opened in the radial and angular center. Following previous observations (Figure 3Bii), we concluded this was the result of the meniscus’ center aligning with the valve opening. If the valve opening been adjusted by a degree to either side, the direction of rotation would be expected to impact Vol_disp_.

Finally, to query whether a reduction in the valve size and inlet channel length would further decrease the Vol_disp_, the total area of the valve was reduced to 50% and 25% of the original dimensions, and this led to volumes of 300 nL ± 58 nL and 210 nL ± 84 nL, respectively (Figure S5). With the valves opened in the radial and angular center, theoretically, reduction in the valve size, combined with adjusting the position of the valve opening, could enable metering to 100 nL or lower. However, the success rate for valve opening decreased to 90% and 70% as the area decreased (to 50% and 25%, respectively); this was not unexpected based on the error associated with the PrTZAL instrument. Alternatively, using the standard valve size but reducing the channel length leading to the valve by 50% allowed for 170 nL ± 53 nL to be metered. This volume was statistically similar to the lowest volume achieved by reducing the valve area to 25% of the original (T-Test: p-value = 0.207, α = 0.05) but with a valve opening success rate of 100%. While this information did not impact architectural changes for applications discussed in this paper, it was useful data to develop a bank of information from which to “tune” this device for future studies.

### An Application in Illicit Drug Titration

The testing of controlled dangerous substances (CDS) in forensic laboratories often requires some amount of the CDS sample to be consumed by presumptive, colorimetric testing to affirm the presence of a drug before an analyst conducts confirmatory testing, such as mass spectrometry. However, there may be some cases in which the collected sample is limited and does not permit both presumptive and confirmatory testing. Here, we show the proof-of-concept application of our titration device to automate the presumptive testing of cocaine while minimizing the amount of diluted cocaine required for such testing.

**Figure 4:**
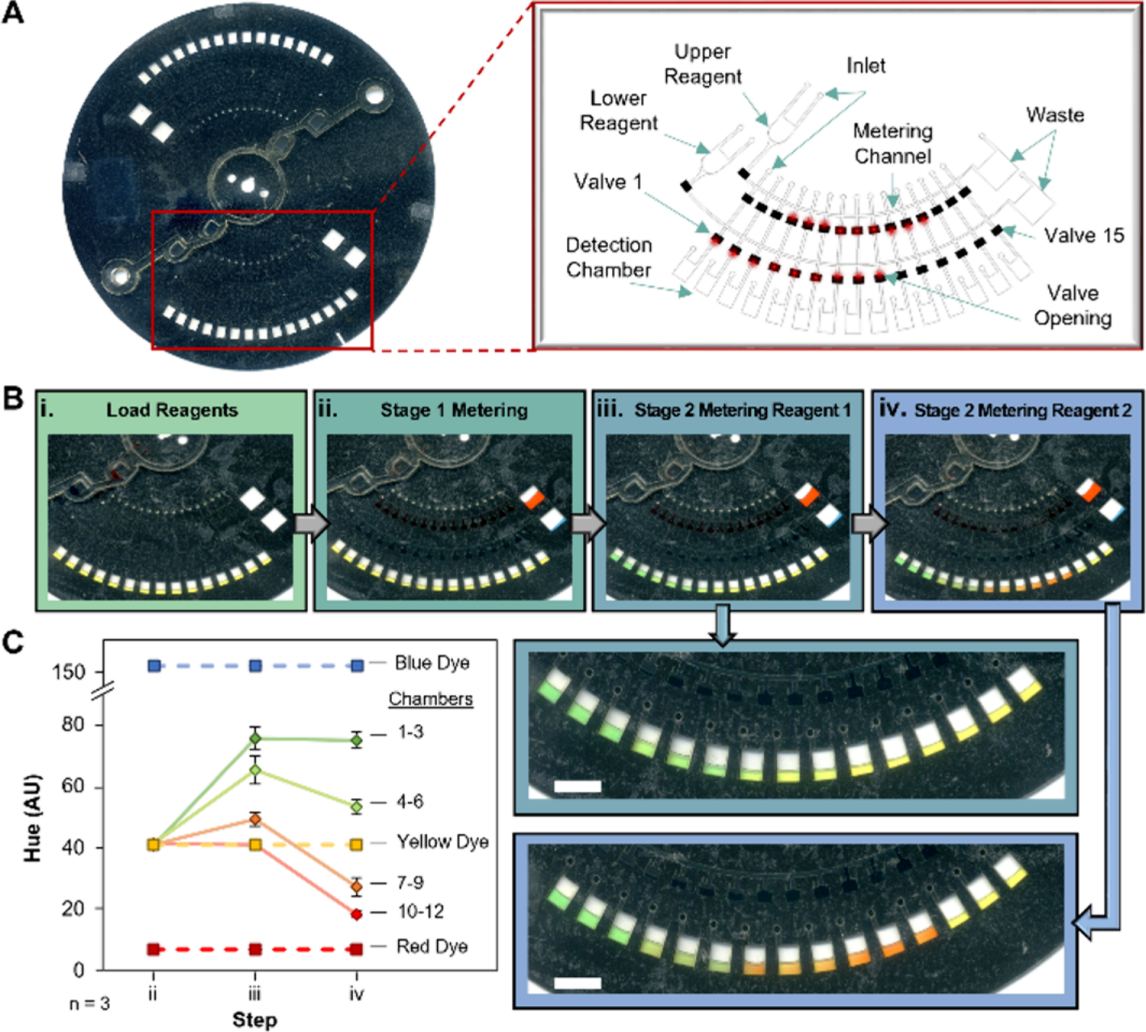
Dye study demonstrating application of two-stage metering principle to colorimetrically detect a titrated analyte. (A) Scanned image of a microfluidic disc for nanoliter metering of two reagents into detection chambers. The inset contains a labeled schematic of a single domain. (B) Dye study demonstrating the workflow implemented for colorimetric detection of cocaine at differing concentrations: (i) dye was pipetted into the disc and (ii) metered to valves; valves containing (iii) blue and (iv) red dye were sequentially opened and rotationally driven into the detection chambers. Zoomed views of the detection chambers following stage 2 metering of reagents 1 and 2 are shown for visual clarity. (C) Analysis of hue changes in the detection chamber during each step of the dye study illustrated in B. Scale bars, 5 mm.

The idea here is to mimic two unit operations, including dilution of the drug and downstream detection via a colorimetric indicator solution. For this application, fluidic architecture was adjusted and a dye study carried out. Figure 4A shows the modified microdisc, which includes a second reagent chamber that also undergoes the two-stage metering process via additional metering architecture, and an additional waste chamber positioned closer to the periphery of the disc. Valves under each reagent chamber were added to control the sequential release of each reagent, and inlets to each detection chamber enabled the addition of preliminary yellow dye for hue difference visualization. The two-stage metering principle with two reagents enabled mixing of the metered reagents in the detection chambers (Figure S6). The detection chamber shape was altered from a circle to a square, large enough to accommodate at least 10 μL, and vents were added to facilitate fluid flow; all other design/architecture remained the same.

Functionality of the modified architecture was assessed using dye studies with red dye representing the diluent, blue dye the presumed illicit drug, and yellow dye the colorimetric indicator. Red and blue dye were loaded into the upper and lower reagent chambers, respectively, with yellow dye pipetted into the detection chambers through the inlet (Figure 4Bi). In stage 1 metering, the valves directly below the reagent chambers were opened and both fluids were metered via centrifugation (Figure 4Bii). Valves containing blue dye were opened according to the Figure 4A exploded view and spun into the detection chamber (Figure 4Biii) prior to opening valves containing the red dye (Figure 4Biv). Valves were opened at the top, middle, or bottom of the two valve arrays to facilitate a titration while maintaining a constant total volume of fluid in the detection chambers. Although color change in the chambers was visually apparent, ambient lighting as well as variability of human interpretation yields a subjective analysis prone to error,^24^ hence, objective image analysis with scanned images was done. Using hue to measure resultant dye in each detection chamber provided empirical analysis that confirmed the visual observations. As blue dye was added to chambers containing yellow dye, hue shifted toward a green color, and as varying volumes of red dye were metered, the hue shifted to five distinct colors (single-factor ANOVA: *p*-value = 1.56 × 10^–10^, α = 0.05) (Figure 4C). Objective volume quantification confirmed chambers 1–3 had a green hue as ∼600 nL of blue dye was added to the yellow dye in the detection chambers. Similarly, chambers 4–6 contained ∼400 nL blue dye and ∼200 nL red dye in the starting yellow dye, resulting in yellow-green, whereas chambers 7–9 contained only ∼200 nL blue dye and ∼400 nL red dye, thus, the final hue was closer to orange-yellow. Finally, chamber 10–12 only had ∼600 nL red dye added to the initial yellow dye, creating a red-orange hue. These findings demonstrate application of the two-stage metering to an adapted disc design where nanovolumes were successfully added to the detection chambers with more consistency than would be possible with manual pipetting.

**Figure 5:**
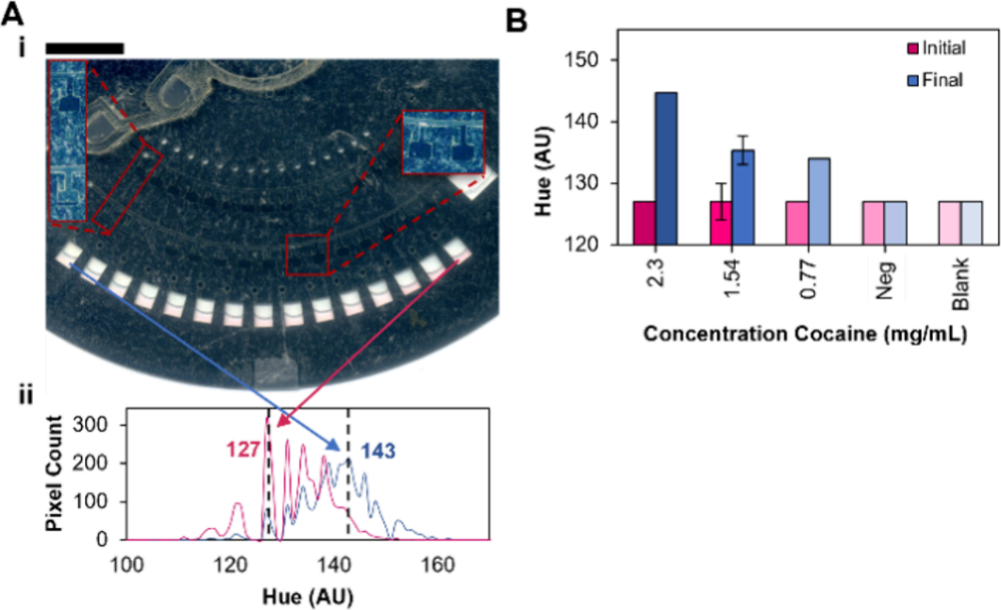
Application of two-stage metering to colorimetrically detect titrated concentrations of cocaine. (A, i) Scanned image of the disc following metering and addition of titrated cocaine standard to a colorimetric indicator, cobalt(II) thiocyanate. Insets show select valves to illustrate the differences in fluid added for each reagent (cocaine and HCl). (ii) Histogram of hue values present in the indicated chambers with the mode shown (dotted line). (B) Results from colorimetric detection of cocaine: the initial hue values when only the colorimetric indicator was present in the detection chambers, and the final hue values upon addition of titrated cocaine standard. Scale bar, 10 mm.

Figure 5 shows utilization of the same disc architecture and experimental workflow as illustrated in Figure 4A-B applied to presumptive testing of a cocaine; here, hydrochloric acid (HCl) is the diluent and Scott’s reagent (cobalt(II) thiocyanate) is a colorimetric indicator used in routine forensic CDS analysis that changes from a pink hue to a blue-colored coordination complex in the presence of cocaine.^26^ The cocaine standard that was originally 1 mg/mL was vacufuged to remove the acetonitrile as working with organic solvents in the microdevice can cause unnecessary complications, and higher concentrations of the drug were preferred for this study. The cocaine standard was reconstituted in HCl to a final concentration of 10 mg/mL; this predilution concentration is relevant, as the threshold for toxic levels of cocaine in the blood is around 1 mg/mL.^27^ The diluent (HCl) was pipetted into the upper reagent chamber, drug to the lower reagent chamber, and cobalt(II) thiocyanate to the detection chambers. Figure 5Ai shows the disc after reagents were metered with exploded views depicting valves opened at differing radial positions and, thus, retaining dissimilar volumes of fluid. When viewing the fluid in the chambers from left to right, the left-most chamber contained the highest volume of cocaine (∼600 nL) with no diluent, thus exhibiting a final blue color with a mode hue of 143 (Figure 5Aii). Moving right, drug added to the detection chambers became more dilute with chambers on the far right representing the blank condition. Results comparing the hue of all detection chambers before and after the drug titration indicate a statistical difference between the initial and final hue (two-factor ANOVA: *p*-value = 2.75 × 10^–9^, α = 0.05) (Figure 5B). Conversely, there was no difference in initial and final hue between the negative controls, which had only diluent metered, or the blank chambers containing only indicator. Additionally, there was a significant difference in the final hue of all concentrations, except for 1.54 and 0.77 mg/mL (Paired T-Test: *p*-value = 0.422, α = 0.05), which was expected as this is the lower limit of analytical testing for the indicator,^26^ and the control conditions. Here, the microfluidic titration of drugs enabled metering of sample in the nanoliter-range with minimal human intervention.

### Nanoliter Metering Disc Customizability

**Figure 6:**
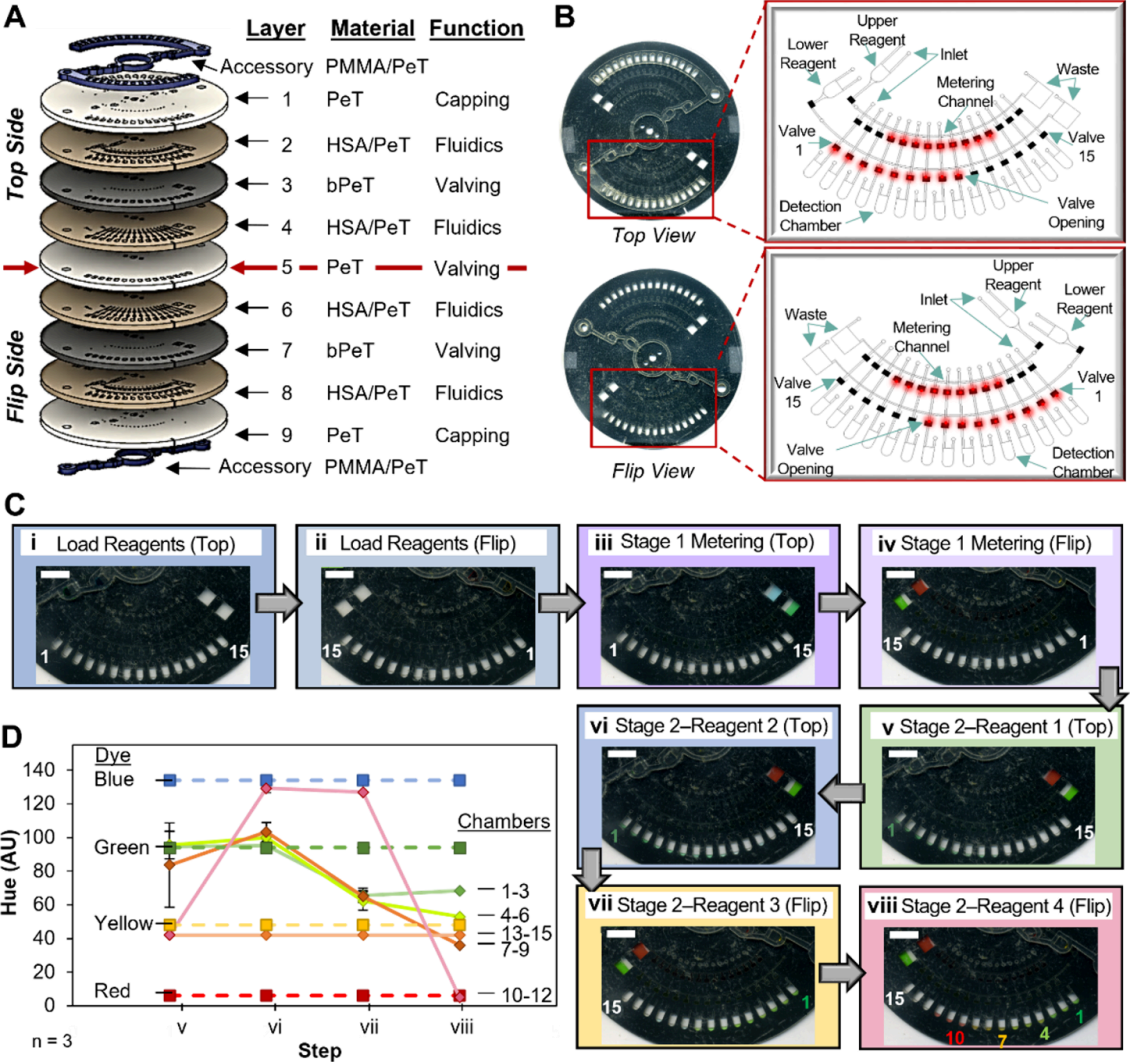
Proof-of-concept demonstration for two-way metering principle on the nine-layer NMD for broader applications. (A) Exploded view of double-sided nine-layer disc. (B) Scanned images of a nine-layer NMD for metering four reagents into detection chambers. The insets contain labeled schematics of the front and back of one domain. (C) Dye study demonstrating the workflow implemented for titration of forward and reverse primers at different concentrations. Reagents were loaded into (i) the front and (ii) back of the microfluidic disc prior to stage 1 metering on the (iii) front and (iv) back. Dye was sequentially metered into the detection chambers beginning with (v) green, (vi) blue, (vii) yellow, and (viii) red dye. (D) Objective measurement of hue changes in the detection chambers during the stage 2 metering of reagents (C,v–viii). Scale bars, 10 mm.

Having demonstrated the applicability of two stage metering on the NMD, it is critical to demonstrate the customizability of the microfluidic architecture to enable a broader range of applications. Increasing the number of reagent chambers enhances the utility for multiplex titration that may be required for more complex reactions. As a proof of concept, and to accommodate the number of chambers necessary to facilitate a multireagent titration without increasing the disc size, additional fluidic layers were added. A nine-layer NMD was PCL fabricated using PeT, bPeT, HSA, and PMMA (Figure 6A). The four disc layers above layer 5 (the “top” side) are a mirror image of the four below (the “flip” side). While not ideal, the added manual step of flipping the disc to access architecture on the flip side effectively doubles the fluidic architecture, and thus, the number of reagents that can be metered while maintaining disc size. Microfluidic layers with laser-cut microchannels (2, 4, 6, and 8) were cut in clear PeT/HSA while the laser-actuated valving was in bPeT layers (3 and 7). Note that in Figure 6A the architectural features on the corresponding layers above and below layer 5 overlap, including the valves; hence, it was critical to establish that opening a valve in layer 3 did not open the corresponding valve in layer 7 to ensure both sides of the disc could be used independently. This valve opening process was depth-specific for two reasons: first, the focal distance between laser of bPET surface was carefully controlled, and second, we believe that having fluid in the valve prior to opening acts as a heat sink, dissipating the thermal energy and preventing the opening of overlapping valves. Bonded to layers 1 and 9 were PMMA accessory pieces that served to increase the volume in the reagent and detection chambers. The accessory PMMA piece on the top side increases the volume of the reagent and the detection chambers, while that on the flip side only encapsulates the reagent chambers. This asymmetrical design was purposeful as the PMMA allowed for easier fluid recovery from the detection chambers via the top side while enabling image analysis to be conducted using scanned images of the flip side without the disruption of shadows from PMMA. The architecture in any single domain on this disc was similar to that used for the drug titration application, with the main difference being that both the top and flip side have architecture for two-stage metering (Figure 6B).

To ensure functionality in the combinatorial titrating of four reagents in parallel into the detection chambers, a dye study was carried out using standard food dyes diluted in water (Figure 6C). As proven in Figure 3, rectangular valves opened at different radial positions (top/center/bottom) provide a predictable metered volume that is delivered to a downstream chamber. To put this into practice, we tested the ability to create a hue gradient across detection chambers by valving at the top, center, or bottom of the valves in the upper and lower arrays, on both the top and flip sides of the disc. The resulting color change in the detection chambers was visually apparent, but objective colorimetric analysis was performed on scanned images of the disc using ImageJ to define hue values for the detection chambers at different steps in the metering process (Figure 6D). Green, blue, red, and yellow dyes, with respective hue values of 94, 134, 48, and 6, were sequentially metered into the detection chambers. With the addition of each dye, the empirical hue value shifted toward that of the stock dye value: chambers containing green dye exhibited a shift in hue from 94 toward 134 as blue dye was added. Similarly, hue decreased toward 48, then 6 as yellow and red dye were added, respectively. Specifically, chambers 1–3 had an estimated 600 μL of green dye and 600 μL of yellow dye added to the detection chamber, without any blue or red dye, creating a yellow-green hue. Chambers 4–6 shifted from green to teal to yellow-green to a dark yellow-green as 400 μL of green dye, 200 μL of blue dye, 400 μL of yellow dye, and 200 μL of red dye was added. Chambers 7–9 had a similar hue shift although the final color was more orange, since 200 μL green dye, 400 μL blue dye, 200 μL of yellow dye, and 400 μL of red dye was added. Chambers 10–12 transitioned from clear to blue to red as 600 μL of blue dye then 600 μL of red dye was added, creating a red-purple hue in the chambers, while chambers 13–15 were a control with only water present. After metering the final dye, the hue in the detection chambers had shifted into five distinct colors (single-factor ANOVA: *p*-value = 7.71 × 10^–10^, α = 0.05). These findings demonstrate that the two-stage metering applied to the nine-layer microfluidic disc, wherein 4 reagents are titrated via 60 valves into 15 detection chambers, provided excellent, autonomous, combinatorial metering with high consistency and precision.

## Conclusions

The optimization of many traditional assays can be time- and labor-intensive, and when that optimization involves sample, two or more reagents, and multiple parallel experiments, this can result in the consumption of copious amounts of the reagents. It is for this reason that the NMD for automated, parallel titration of reagents using nanovolumes was developed and described here. Unlike similar existing devices,^2,4,18^ this rotationally driven disc was fabricated from polymeric material using the PCL fabrication method^21^ that is ideal for rapid, iterative prototyping with cost-effective materials, costing < $1 USD per disc and consuming less than 30 min to fabricate. The desire to have this be an autonomous system (once loaded) required a simple method for fluidic valving, and the laser-actuated valving approach exploiting black PeT functioned superbly in this role. However, what we describe here goes beyond simple opening of fluidic circuits. The laser valves have been used in a number of applications^23,28−32^ where the rectangular shape and size was purposeful for three reasons: 1) the 2D size (width, height) presented a large target for repeatable laser ablation of an 80 μm opening, 2) the rectangular shape (width > height) compensated for the slightly poorer accuracy in finding the exact angular coordinate (defined by the motor), and 3) after opening, the 1.5 mm × 2.5 mm × 0.1 mm valve typically retained less than 1 μL, a negligible volume of fluid relative to what was ported through the valve. However, it became clear that treating the valve less as transitory architecture and more like a chamber offered the potential for a new functionality–metering.

Ignoring its “pass through” function and focusing on metering, the dimensions and shape of the valve become important, and initial proof-of-principle experiments demonstrated two-stage metering. The imparted architectural features enable tunability for stage 1 aliquoting into the valves, while the laser-ablated opening permits selective valving for stage 2. With this method, three variables contributed to the volume of fluid dispensed into detection chambers: (1) the valve shape, (2) the radial and angular positioning of the laser ablated opening in the valve, and (3) the architecture feeding fluid into the valve. We methodically evaluated how these three variables affected the volume of fluid metered in the second stage, showing that circular and triangular valves lacked reproducibility in terms of opening the valve, while the rectangular valves were effective for controlled metering of fluid defined by a 100% success rate for valve opening at a specific position over hundreds of runs. Using the same radial and angular coordinates for hitting each of the top, middle, and bottom positions of the rectangular valves, shows excellent accuracy and precision, translating directly to the second metering stage where <200 nL volumes could be reproducibly metered. It is important to mention that our standard deviations are comparable to those observed when manually pipetting nanovolumes. For example, a 0.1–2.5 μL Eppendorf pipet reports systematic measurement errors of ±12% when pipetting 250 nL. Our system similarly reports a ± 12.5% error when valving for 200 nL on the NMD.^33^

We demonstrated proof-of-feasibility with the design of a microfluidic disc for assay optimization of a colorimetric reaction using a 16-chamber disc designed for presumptive testing of cocaine. Not only was the automation successful, but the advantage offered by reduced reagent volumes was obvious, along with a substantial reduction in sample consumption. In theory, this disc design could be expanded to empirically define the analytical range of a colorimetric indicator by increasing the number of metering and detection chambers and using nanovolumes of reagents that cannot be pipet by hand. Alternatively, detection chambers could be preloaded with a variety of colorimetric indicators for suspected CDS (e.g., Marquis Reagent, Mandelin Reagent, etc.) to test for mixtures of illicit drugs or permit one disc to test for a variety of them.

To demonstrate the effectiveness of this approach for further applications, a 9-layer disc was fabricated containing 15 detection chambers in each of the two domains, with a “flip” being required to access the reagents metered on the lower half of the disc. While the required manual flipping step is unfavorable, the inclusion of identical architecture on the back of the disc permitted four dyes to be titrated into the detection chambers, suggesting functionality to perform more complex reactions.

The limitations imposed by the PrTZAL system impact the accuracy and precision of the radial and angular positioning of the laser-ablated holes in the sacrificial valves. While this error is relatively small and still enables nanoliter volumes to be metered, the relative standard deviations associated with the Vol_disp_ can be as high as 20% of the total Vol_disp_. It is worth noting that the described PrTZAL system is a first generation prototype instrument that did not require a high level of precision in previous applications where a valve acted as transitory architecture. However, upon the demonstration purported here that these laser-actuated valves could function as a chamber for metering nanovolumes, it has become clear that improvements can be made to the PrTZAL system to enhance the accuracy and precision of laser-ablated valve openings. Accordingly, we intend to modify the second generation of the PrTZAL system to minimize the radial and angular error associated with valving by sourcing hardware (e.g., stepper motors and linear actuators) with smaller inherent error. We believe this will improve the accuracy and precision of the instrument, decreasing the relative standard deviations associated with the Vol_disp_ and possibly enabling smaller volumes of fluid to be metered.

Overall, this inexpensive microfluidic disc enables nanoliter volume of fluid, between 150 nL and 1 μL, to be metered and manipulated with minimal manual intervention. This platform can conduct multiple reactions in parallel in an automated, cost-effective manner that removes the human variability and error associated with standard pipettes, and it does so using a fraction of the volume of reagents needed for in-tube (or in-well) assay optimization. The NMD can be modified to fit a variety of applications that require assay optimization of multivariate parameters, saving scientists copious amounts of time, effort, and resources.

## Supporting Information Available

Additional experimental details and methods, including the external system used, experimental theory, and evaluation of valve parameters (PDF)
