# Supplementary material for: Automated Nanoliter Volume Assay Optimization on a Cost-Effective Microfluidic Disc
Source: Anal Chem. 2024 Dec 28;97(1):300–11. doi: 10.1021/acs.analchem.4c04210 (PMC11740179; doi:10.1021/acs.analchem.4c04210)
Supplement: Supplementary file 1 — ac4c04210_si_001.pdf [file ac4c04210_si_001.pdf]

SUPPORTING INFORMATION

AUTOMATED NANOLITER VOLUME ASSAY OPTIMIZATION ON A COST-EFFECTIVE MICROFLUIDIC DISC

Renna L. Nouwairi,[a] Carter K. Jones,[a*] Maura E. Charette,[a] Emilee Holmquist,[a] Zoey Golabek,[a] James P. Landers[a,b,c]

[a]Department of Chemistry, University of Virginia, Charlottesville, Virginia 22904, USA

[b]Department of Mechanical Engineering, University of Virginia, Charlottesville, Virginia 22904, USA

[c]Department of Pathology, University of Virginia, Charlottesville, Virginia 22904, USA

*Corresponding Author: Carter Jones (ckj2sc@virginia.edu)

Table of Contents:

Figure S1: Mechatronic spin system for performing microfluidic unit operations on the centrifugal microdevices.

Figure S2**:** Dye study for the two-way metering principle.

Figure S3: Instrumental accuracy and precision.

Figure S4: Aberrant results in the laser ablation valving process.

Figure S5: Evaluation of different metering architectures.

Figure S6: Two-stage metering theory for multiple reagents**.**

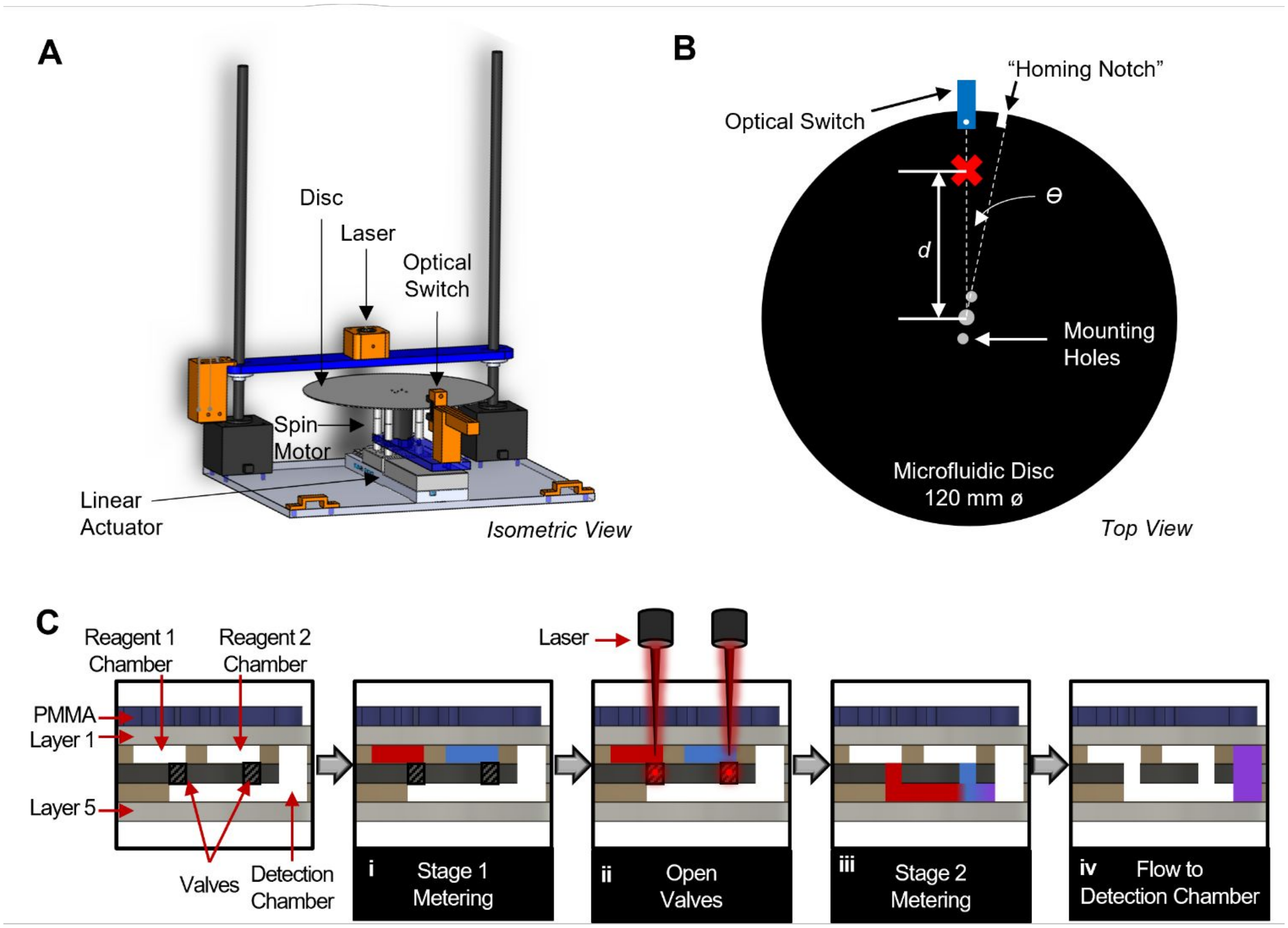

Figure S1. Working principles of the power, time, z-height actuated laser (PrTZAL) system and active valving method implemented. (A) The custom-built mechatronic PrTZAL system that accurately positions the laser over a valve by spinning a disc mounted to a linear actuator that moves in the x-direction (adapted from Woolf *et al*. (*23*)). (B) Diagram showing a top view of a microfluidic disc containing holes to mount the disc to the PrTZAL system. The red "X" represents a valve set a distance (*d*) from the center of rotation at an angular distance (*Θ*) form a homing notch. The photointerrupting optical switch enables the system to spin the disc to a set angle as defined by the homing notch while the linear actuator radially positions the disc relative to the laser. (C) A side view of the 5-layer microfluidic disc depicting the dual-stage metering workflow, including (i) loading and aliquoting reagents, (ii) opening the normally-closed valves via laser ablation, (iii) spinning fluid through the valve to the (iv) detection chamber.

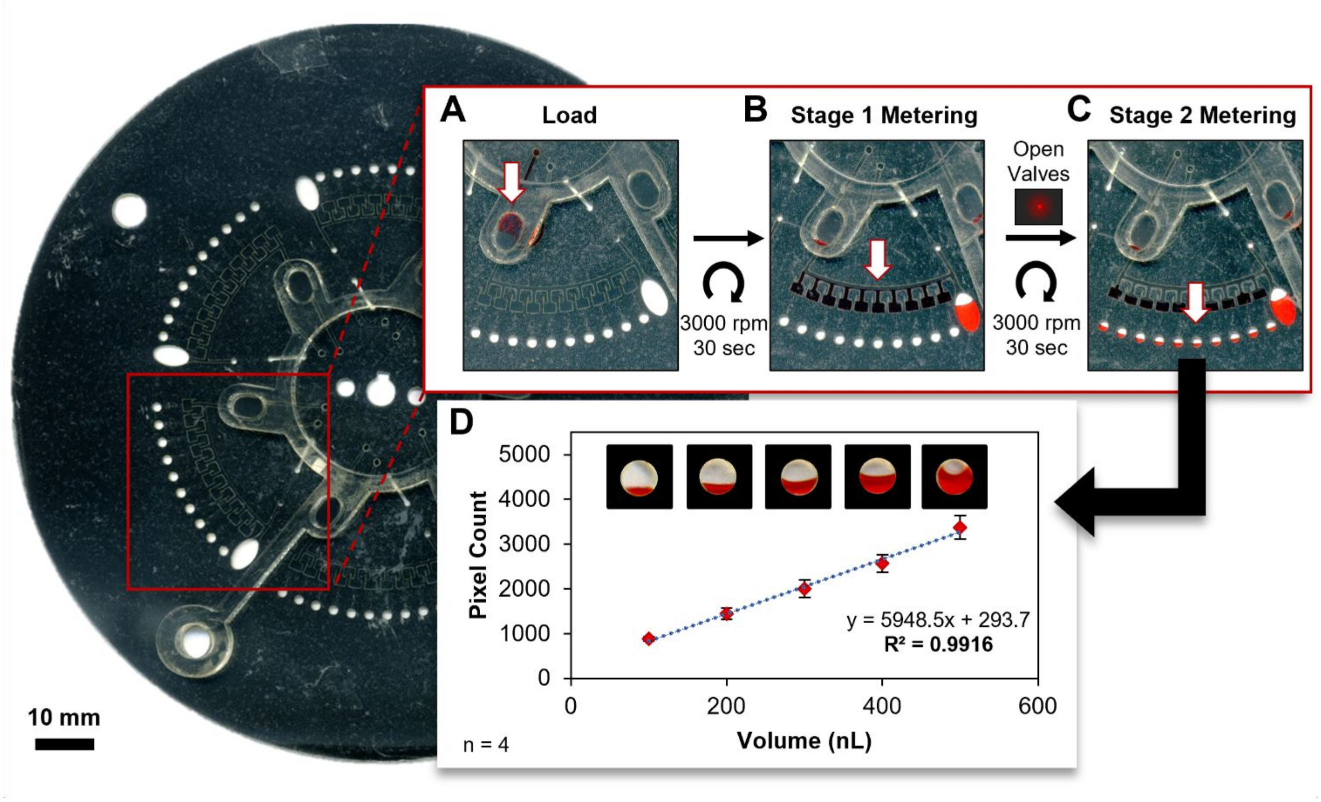

Figure S2. Scanned image of the fully assembled centrifugal microfluidic disc and a dye study illustrating the two-way metering working principle. The inset depicts the nanoliter metering workflow, including (A) loading fluid into the reagent chamber, (B) stage 1 metering, whereby fluid is aliquoted from the reagent chamber into the valves, and (C) stage 2 metering of fluid into the detection chamber post-valve opening. (D) A calibration curve correlating pixel count to a known volume for objective determination of fluid metered into the detection chambers. The results indicate a linear trend (y = 5948.5x + 293.7; R^2 = 0.9916), and subsequent work extrapolated dispensed volumes from this trendline.

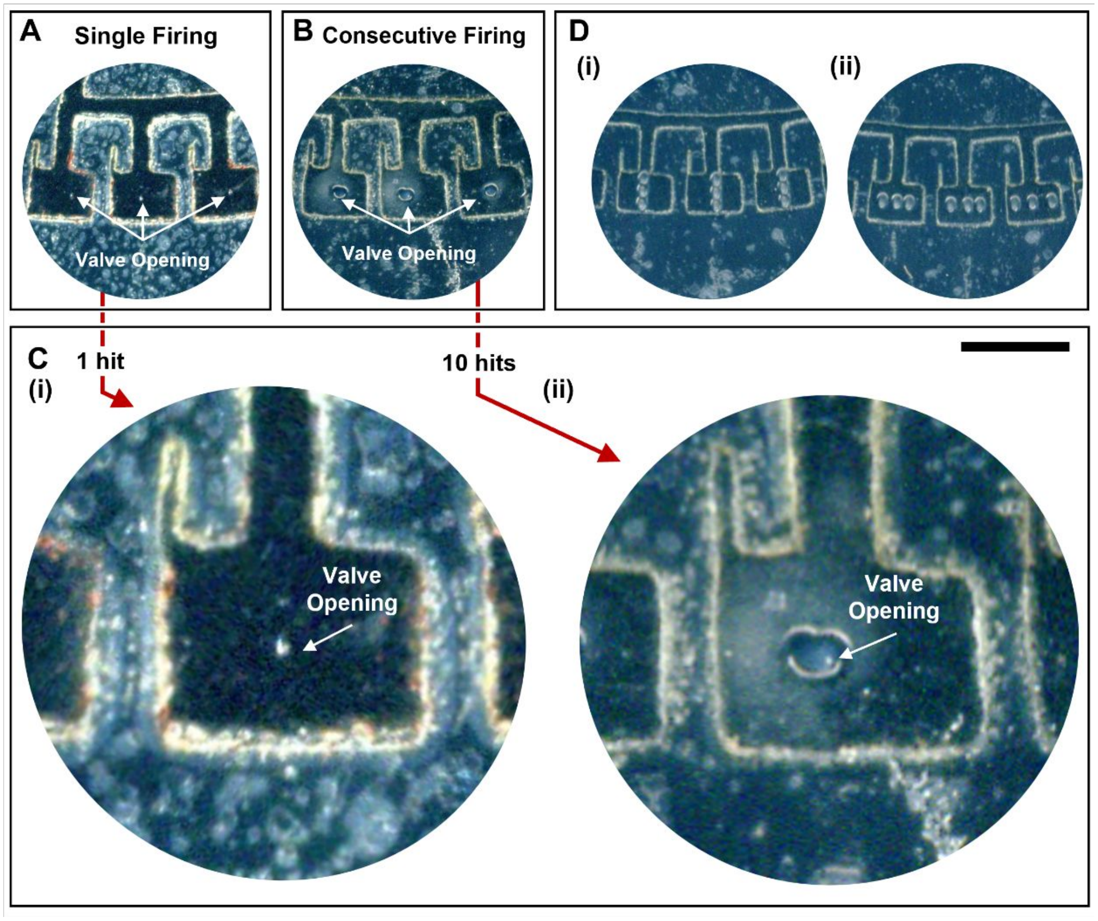

Figure S3. Scanned images of valves after single and consecutive opening(s) to assess instrumental accuracy and precision. (A) Valves opened with a singular laser ablation. (B) Valves opened consecutively (n=10) at the center radial and angular position. (C) Enlarged images highlighting the accuracy and precision of opening a valve (i) once or (ii) 10 consecutive times. (D) Consecutive valve openings (n=10) at varying (i) radial and (ii) angular positions. Scale bar, 1 mm.

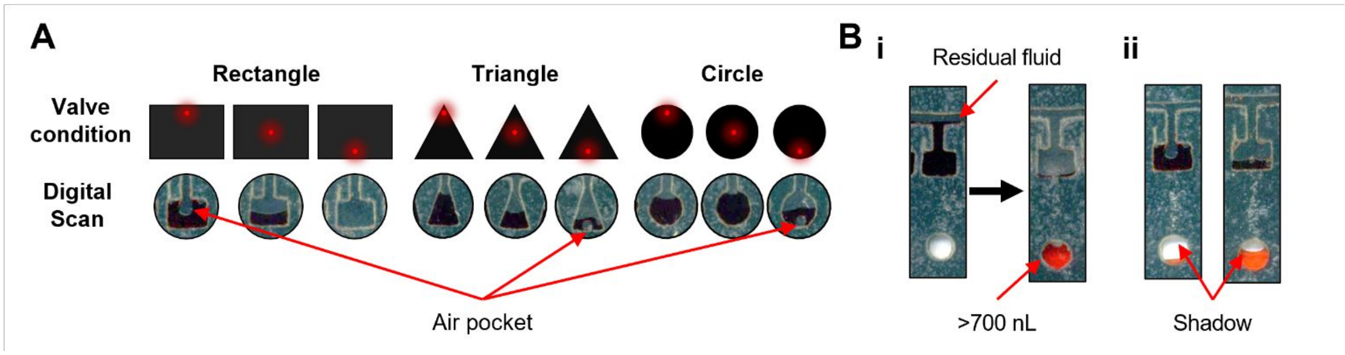

Figure S4. Representative images illustrating conditions that caused aberrant results. (A) Scanned images of valves opened at varying laser positions with arrows indicating air pockets created by laser ablation that trapped fluid in the valves. (B) Two conditions responsible for overestimation of fluid in the detection chamber, including (i) residual fluid in the metering channel that caused the final volume to exceed 700 nL, and (ii) shadows in the detection chambers resulting from scanned images.

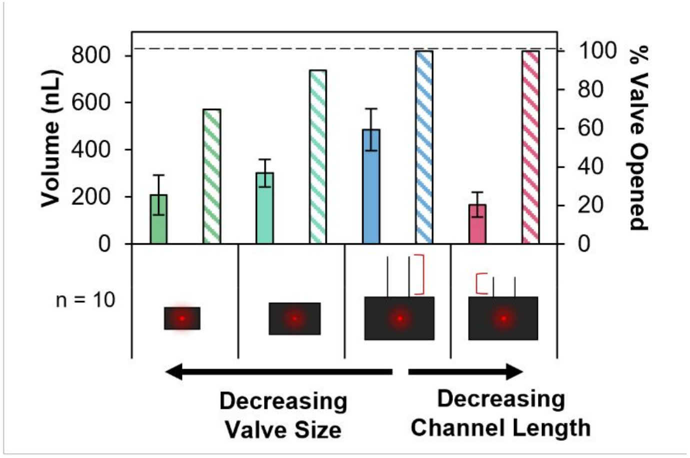

Figure S5. Demonstrating different strategies to adjust the final metered volume. Changes in volume as a result of reducing the valve size compared to reducing the length of the channel leading to the valve. The original valve size (blue bars) was reduced to half (teal bar) and a quarter (green bar) of the original area, and the length of the metering channel leading to the valve was reduced by half (pink bars). The striped bars represent the percent of successful valve openings.

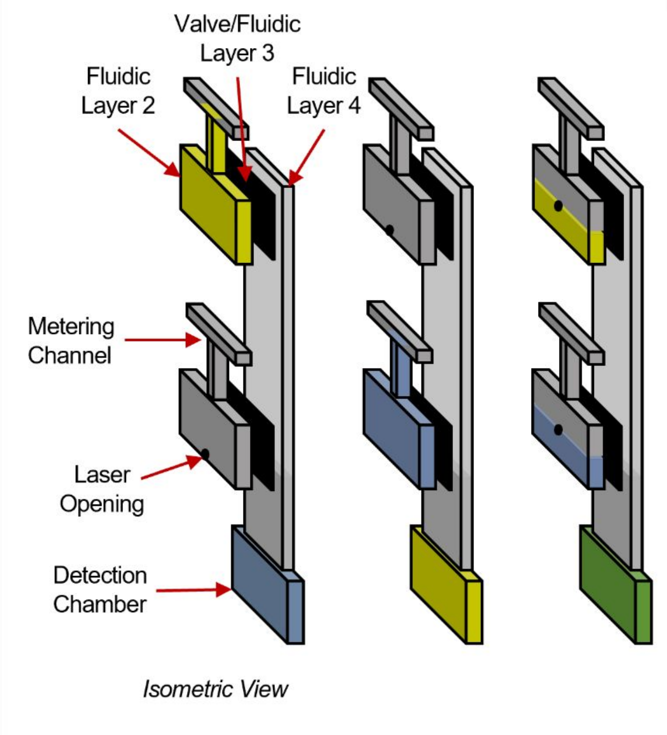

Figure S6. Two-stage metering principle for two reagents on the microdevice. After sample loading, stage 1 metering aliquots reagents into fluidic layer 2. The valves in layer 3 are selectively opened to tune the dispensed volume through the laser opening in stage 2 metering. The reagents are then spun into and mixed in the detection chamber.
